# Adipose-derived stem cell exosomes: emerging roles and therapeutic application

**DOI:** 10.3389/fphar.2025.1637342

**Published:** 2025-09-25

**Authors:** Yuhan Gong, Hui Ma, Zhongren Zheng, Xinjie Wang, Jiahua Zhang, Xiaowei Zhao

**Affiliations:** ^1^ Department of Clinic Medicine, Jining Medical University, Jining, Shandong, China; ^2^ Department of Joint and Sports Medicine, Affiliated Hospital of Jining Medical University, Jining, Shandong, China

**Keywords:** adipose-derived stem cells, exosomes, tissue regeneration, cell-free therapy, regulatory pathway

## Abstract

Stem cell-derived exosomes have broad application prospects in different medical fields, and are increasingly being considered a replacement for Mesenchymal stromal cells (MSCs) therapy. Adipose-derived stem cells (ADSCs) are an efficient and high-quality source of stem cell exosomes because ADSCs can be easily obtained from autologous adipose tissue and there are only minor ethical concerns, also ADSCs shown multipotent differentiation potential, self-renewal potential, low immunogenicity, and high proliferation rate. Exosomes derived from ADSCs have the function of promoting tissue regeneration through activation or inhibition of multiple signaling pathways (such as Wnt/βcatenin, PI3K/Akt), and immunomodulation, angiogenesis, cell migration, proliferation and differentiation, and tissue remodeling. This review presents the current state of knowledge on ADSCs exosomes and summarizes the use of ADSCs exosomes in stem cell-free therapies for the treatment of diabetes mellitus, cardiovascular, wound healing, neurodegenerative, skeletal, respiratory diseases, and skin aging and other conditions, thus providing novel insights into the clinical applications of MSC-derived exosomes in disease management.

## 1 Introduction

### 1.1 MSCs sources and characteristics

MSCs are a group of multipotent progenitor cells found in a number of tissues such as bone marrow and adipose, which can be isolated from adult and foetal tissues. MSCs show promising applications in clinical tissue regeneration, as they have the ability to proliferate and differentiate into a variety of cell lineages, such as into adipocytes, chondrocytes, and osteoblasts, as well as hepatocytes and neuronal cells (Kariminekoo et al., 2016; Crapnell et al., 2013).

### 1.2 Limitations of MSCs therapy

Initial studies suggested that MSCs may play a key role in tissue repair, but with continued research it has been found that the low survival rate and low engraftment potential of MSCs in damaged tissue regions limit the effectiveness of MSCs in tissue repair (Hade et al., 2021). It was later shown after several studies that the reparative effect of MSCs in injured tissues does not depend on their implantation and differentiation at the site of injury, but on their paracrine activity (Barreca et al., 2020). MSCs can produce and release a variety of growth factors, chemokines and cytokines. These factors can reduce apoptosis and tissue fibrosis, stimulate extracellular matrix remodelling, inhibit local inflammation, regulate immune responses, induce regeneration, salvage damaged cells, reduce tissue damage, and ultimately accelerate organ repair through paracrine effects (Keshtkar et al., 2018). However, safety concerns regarding MSCs persist. These include a potential risk of malignant transformation due to the *in vitro* expansion required for therapy, as well as the ability of allogeneic MSCs to partially evade immune recognition. In addition, MSCs have the potential to trigger adaptive immunity and compromise efficacy; MSCs can modulate the immune system by being phagocytosed by antigen-presenting cells (APCs), and subsequent MSC antigens presented by APCs result in decreased anti-inflammatory activity and therapeutic efficacy. On the other hand, recognition and clearance of MSCs by the host immune system may also limit the duration and possible efficacy of treatment for many MSCs (Caplan et al., 2019). Through further research, it was found that MSCs are able to release a large number of extracellular vesicles (EVs), which can carry the active factors produced by MSCs to deliver information to damaged cells or tissues to participate in tissue regeneration, exerting biological activities similar to those of MSCs (Rani et al., 2015).

### 1.3 Extracellular vesicles biology and classification

Extracellular vesicles (EVs) are membrane-enclosed nanoscale particles released from cells. Almost all cells, both prokaryotic and eukaryotic, release EVs as part of their normal physiology and in response to pathological conditions (O'Brien et al., 2020). EVs are a general concept that can be further broken down into different categories based on the size of EVs, including apoptotic vesicles (1,000–5,000 nm), microvesicles (100–1,000 nm), and exosomes (30–200 nm).

### 1.4 Role of paracrine factors and exosomes

Exosomes carry various biomolecules, including proteins, lipids, and nucleic acids, from their parent cells. Exosomes transmit information in the extracellular space, altering the biological response of the recipient cell by delivering proteins, nucleic acids, and other substances that can inhibit disease but may also promote it (Kalluri and LeBleu, 2020). Based on this property of regulating intracellular pathways, exosomes play a potential utility in the treatment and control of many diseases, including neurodegenerative diseases and tumour therapy (Younas et al., 2022; Zhang and Yu, 2019). In regulating the tumour microenvironment and promoting tumourigenesis, exosomes have potential functions related to immunomodulation and have been implicated as carriers of anti-tumour immune responses (Urabe et al., 2020; van Niel et al., 2018).

### 1.5 Exosomes derived from adipose-derived stem cells

Adipose-derived stem cells(ADSCs) have received widespread attention due to their multifaceted, abundant, and easily isolated characteristics. A large number of studies have demonstrated that ADSCs are promising for a wide range of applications in regenerative medicine. ADSCs are an important stem cell type isolated from adipose tissue with properties of multidirectional differentiation and self-renewal (Deng and Chen, 2022). Increasingly, research is focusing on exosomes derived from adipose-derived stem cells (ADSCs-exos). ADSCs-exos promote tissue regeneration, immunomodulation, angiogenesis, cell migration, proliferation, differentiation, and tissue remodeling by modulating multiple signaling pathways (Li Y. et al., 2023). This paper reviews the current state of research on ADSCs-exos and summarises the application of ADSCs-exos in stem cell-free treatment of diabetes, cardiovascular, wound healing, neurodegenerative, skeletal, respiratory disorders, and skin ageing, thus providing new insights into the clinical application of ADSCs-exos in disease management.

## 2 Adipose tissue as a source of adult stem cells: functions and advantages

**FIGURE 1:**
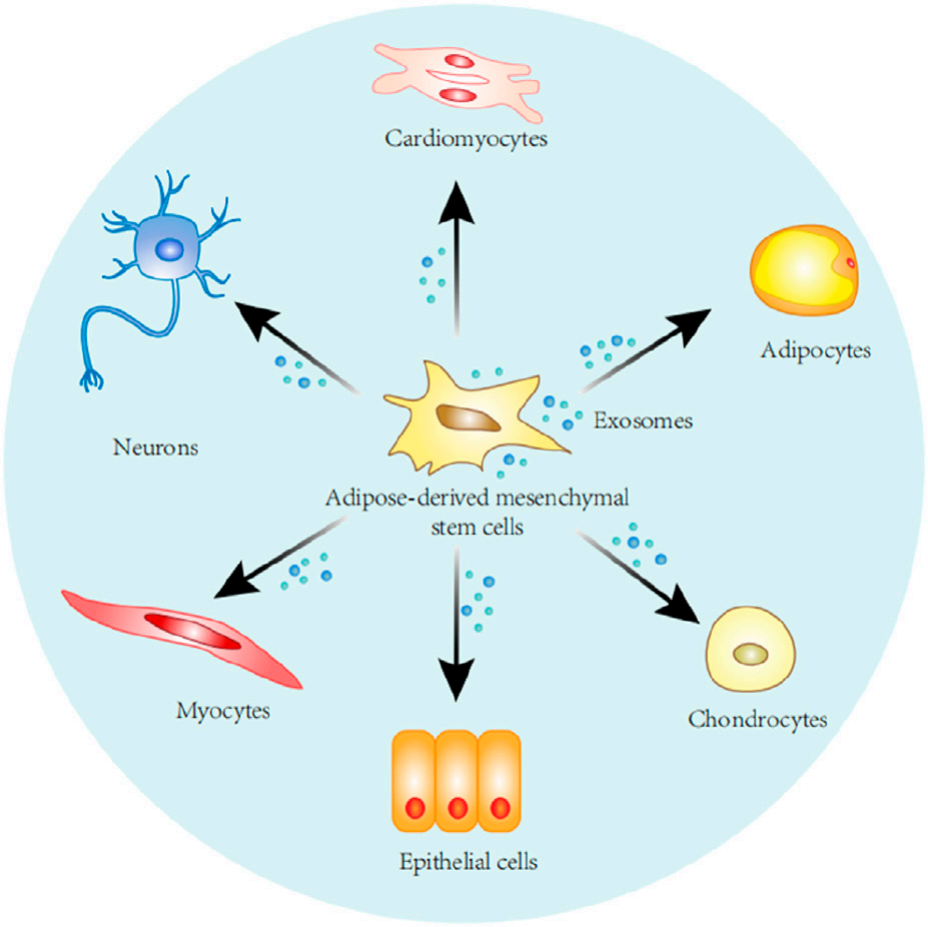
Stem cells derived from adipose tissue can self-renew and differentiate along various cell lines, including adipocytes, chondrocytes, epithelial cells, myocytes, nerve cells, cardiomyocytes.

Stem cells are undifferentiated cells characterized by their extensive proliferative and self-renewal capacities, as well as their potential to differentiate into various cell types and tissues (Kolios and Moodley, 2013). Stem cells are capable of differentiating into different cell populations including adipocytes, osteoblasts, myoblasts, and chondrocytes (Figure 1), which can act as a repository of cells to maintain, repair, and regenerate different tissues (Rodriguez et al., 2005; Laplane and Solary, 2019). Stem cells are divided into three main categories, namely embryonic stem cells, adult stem cells and induced pluripotent stem cells. Although embryonic stem cells exhibit unlimited differentiation potential both *in vitro* and *in vivo*, their clinical application is constrained by ethical, legal, safety, and efficacy concerns. Stem cells from adult tissues can circumvent these issues, and although adult stem cells can only differentiate into a limited number of cell types, these cells can be used in an autologous form, overcoming ethical and legal issues (Gimble and Guilak, 2003; Bacakova et al., 2018). Adult stem cells are undifferentiated cells residing in various tissues throughout the body. Typically maintained in a quiescent, non-dividing state, they can be activated to proliferate and differentiate following tissue damage or cell death to replace lost cells. Due to this proliferative property and the ability to regenerate tissues, adult stem cells have the potential to treat various degenerative diseases as well as aging (Cable et al., 2020).

Adipose tissue is usually considered to be inert in terms of biological activity, but with research it has been found to be rich in stem cells. Adipose tissue-derived adult stem cells are Mesenchymal stromal cells found in the subcutaneous tissue at the base of the hair follicle (dermal papilla cells), the dermis (dermal layer cells), the interfollicular dermis, and subcutaneous dermis (Bunnell, 2021; Mazini et al., 2020). The main advantages over other adult stem cells include abundant source of adipose tissue, easy tissue collection and cell isolation for extraction (Banas et al., 2007). Based on these advantages make ADSCs one of the most favourable stem cells for clinical cell therapy (Mazini et al., 2020). After a number of studies, it has been shown that the differentiation of ADSCs is not limited to the adipocyte lineage, but is capable of differentiating into a variety of cell lineages or a variety of cells of mesodermal origin including chondrocytes, osteoblasts, and adipocytes under appropriate stimulation *in vitro* (Gimble and Guilak, 2003; Mazini et al., 2020; Veronesi et al., 2014). ADSCs cultured under horse serum conditions can express myosin light chain kinase (a protein marker for skeletal muscle cell lineage), which provides a research idea for ADSCs to differentiate skeletal muscle cells for repairing damaged skeletal muscle (Gimble and Guilak, 2003).

Adult stem cells can be isolated from various tissues and their regenerative potential has been widely noted, so researchers need to conduct comparative studies on adult stem cells from different tissues (Heo et al., 2016). The first application for cell therapy was bone marrow stem cells, but bone marrow is relatively difficult to obtain, and it can be traumatic for patients during bone marrow harvesting, and the number of stem cells extracted from bone marrow is small, and it is often necessary to undergo *in vitro* expansion to increase the number of cells. Numerous studies have demonstrated that ADSCs can be an alternative to bone marrow stem cells. The ideal source of adult stem cells needs to be easily accessible and capable of extracting a certain number of stem cells, and ADSCs meet these requirements (Banas et al., 2007; Veronesi et al., 2014; Zuk et al., 2001). Treatment by osteogenic differentiation medium induced ADSCs to differentiate towards osteogenesis, exhibiting upregulation of osteogenesis-specific genes and mineralisation, suggesting that ADSCs cells have the potential to promote skeletal recovery (Im, 2013). ADSCs were demonstrated to reverse wall thinning in areas of myocardial scarring in studies performed from rats. Regeneration of cardiomyocytes and blood vessels was found after stem cells were cultured into monolayers and transplanted into rats with infarcted myocardium (Miyahara et al., 2006). It has also been found that these stem cells can improve ventricular function through growth factor-mediated paracrine effects (Madonna et al., 2009). Chandra et al. found that islet-like cell aggregates expressing pancreatic hormones were generated from stem cells cultured from mouse epididymal fat pads and transplanted intraperitoneally into STZ-induced diabetic mice, and blood glucose levels of the diabetic mice returned to normal within 2 weeks (Chandra et al., 2009). Yu et al. found that ADSCs can improve insulin sensitivity by reducing chronic inflammation in diabetic patients, which in turn delays or treats diabetic complications (Yu et al., 2019). Sen et al. found that therapeutic diabetes medications can alter the differentiation pathways of ADSCs, which can respond to the individual’s efficacy of diabetes medications. This ability to predict the efficacy of diabetes treatments suggests that ADSCs can be used as a cell-based biomarker. However, there are fewer studies of ADSCs as biomarkers responding to drug efficacy, and additional experiments are needed to confirm this (Sen, 2019). Adenovirus, herpes simplex virus, lentivirus and retroviral vectors are all capable of infecting ADSCs cells *in vitro*, and the question of whether ADSCs can serve as cellular vectors for the delivery of genes has gradually attracted the attention of researchers. The basic fibroblast growth factor (bFGF) gene was introduced into ADSCs cells and peak secretion of bFGF protein was observed 3 days after viral transduction. This study validated the conjecture of ADSCs as gene carriers, but further studies are needed for the clinical application of ADSCs as gene carriers (Gimble and Guilak, 2003; Katz et al., 1999). The above studies have shown that ADSCs have the ability to differentiate into a variety of cell lines, which are differentiated into specific tissue cells through *in vitro* culture under certain conditions, and that injection of these treated ADSCs into organisms can promote cell regeneration and tissue repair. In addition, ADSCs can be taken from autologous bodies and applied to themselves, avoiding legal and ethical issues, and these features and advantages make ADSCs have an extremely wide range of applications in various clinical disciplines.

## 3 Overview of adipose-derived stem cell exosomes: exosome biogenesis, secretion and uptake isolation of exosomes

Recent studies have increasingly focused on the role of exosomes in health and disease, which play an important role in promoting human health and treating diseases, including the promotion of organismal development, immune response, and tissue homeostasis, as well as the treatment of cardiovascular diseases, neurodegenerative diseases, and cancers (Pegtel and Gould, 2019). ADSCs-exos have been considered a versatile therapeutic tool for the treatment of tissue damage (Xing et al., 2020a). A large number of studies have confirmed that ADSCs-exos promotes damaged tissue repair and that ADSCs-exos can be used as a stem cell-free therapeutic tool to regenerate different tissues such as bone, cartilage, nerves and skin tissues (An et al., 2021; Zhang S. et al., 2018; Feng et al., 2019; Lu et al., 2019).

Exosomes are nanoscale membrane-bound vesicles secreted by donor cells that are able to participate in intercellular transport of substances (Liang et al., 2021). Exosomes contain biomolecules such as proteins, lipids, nucleic acids, and metabolites. By passing these biomolecules between cells the recipient cell undergoes relevant biological reactions (Kalluri and LeBleu, 2020). To further understand how exosomes are involved in intercellular communication mechanisms, a large number of experiments have been performed to study the processes of exosome biogenesis, secretion and uptake (Pegtel and Gould, 2019). Currently, the prevailing view is that the biogenesis of exosomes is as follows: the cell membrane invaginates to form endosomes, endosomes form intracellular multivesicular bodies (MVBs) through secondary invagination, and finally the MVBs fuse with the cytoplasmic membrane and secrete the intraluminal vesicles (ILVs) within the MVBs outside the cell by cytotoxic secretion into exosomes (Kalluri and LeBleu, 2020). The process of exosome biogenesis is divided into four main steps, including the sorting of content uptake, the formation and maturation of MVBs, the transport of MVBs, and the fusion of MVBs with the plasma membrane (Han et al., 2022). It follows that the process of exosome biogenesis is realised mainly around MVBs, which are special intracellular body compartments enriched with ILVs, and MVBs are able to segregate specific proteins, lipids and other cytoplasmic components (Gurung et al., 2021). The general process of MVBs formation is the internalization of extracellular proteins, lipids, small molecule free substances and other components into the cell through plasma membrane invagination to form cytoplasmic membrane bud vesicles, which are called early sorting endosomes (ESEs), which in turn fuse with secreted vesicles such as the endoplasmic reticulum (ER), the trans Golgi body (TGN) and the mitochondria to form late sorting endosomes (LSEs). Subsequently, the LSE plasma membrane invaginates twice to form many ILVs, and the LSEs carrying ILVs are then called MVBs (Krylova and Feng, 2023). Rab5 and its effector VPS34/p150 have been found to be key regulators of ESE to LSE conversion in the plasma membrane. It acts by recycling extracellular material to the cell membrane within minutes after the ESE begins to form ILVs through inward membrane outgrowth, which ultimately results in the segregation and distribution of the contents into vesicles (Mashouri et al., 2019). When the LSE membrane is invaginated, LSE inclusions are mixed and encapsulated to form multiple intraluminal vesicles. Depending on the amount of invagination, this process produces ILVs with different inclusions and different sizes. The remaining membrane of the invaginated LSE membrane acts as an outer membrane, concentrating the formed ILVs within the LSE lumen, a process that leads to further formation of MVBs in the LSE (Gurunathan et al., 2021). Formation of MVBs is at the centre of exosome biogenesis, where ILVs production and membrane outgrowth are key to exosome production (Hurley et al., 2010). It was found that the formation of MVBs and ILVs can be driven by the endosomal sorting complex required for transport (ESCRT), which consists of the ESCRT-0, -I, -II, -III subcomplexes and the atpase VPS4. Among them, the ESCRT subcomplexes jointly promote the formation of MVBs and ILVs through different functions. This ESCRT-driven formation of MVBs and ILVs is an ESCRT-dependent pathway, and ESCRT-dependent pathways mainly include: the Alix-dependent pathway, HD-PTP-dependent pathway and other pathways (Han et al., 2022; Colombo et al., 2014). In addition, MVBs and ILVs can also be formed independently of ESCRT-driven formation. For example, ILVs can be formed in the absence of ESCRT by sorting through the four-spanning protein CD63, which is an ESCRT-independent mechanism. ESCRT-independent pathways mainly include:The nSMase2-ceramide-dependent pathway, Caveolin-1, Flotillins, and Cholesterol (Han et al., 2022; Stuffers et al., 2009; Edgar et al., 2014). Therefore, it is currently believed that MVBs and ILVs can be formed through the ESCRT-dependent pathway and the ESCRT-independent pathway, which may be related to the classification of cellular contents (Carayon et al., 2011).

**FIGURE 2:**
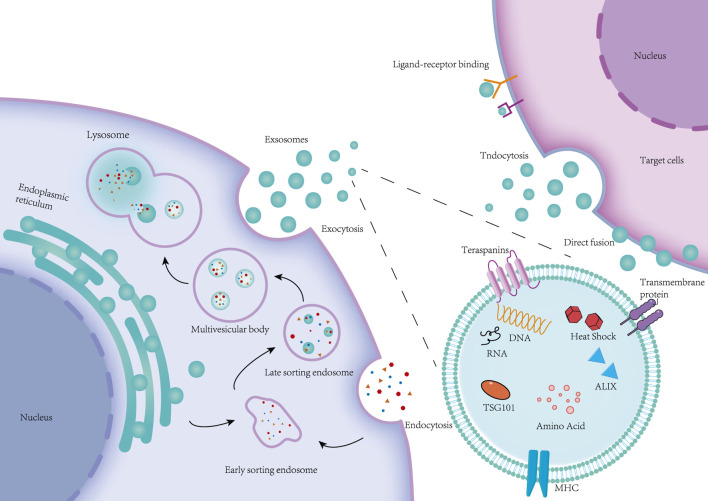
Cell membrane invagination endocytoses some extracellular proteins, lipids, small molecules and cell surface proteins to form vesicles, namely early sorting endosomes (ESE). ESE fuses with vesicles secreted from the Golgi and endoplasmic reticulum to form the late sorting endosome (LSE). The LSE reorganizes its contents through membrane invagination to form a collection of luminal vesicles (ILVs) of different sizes, which are multivesicular bodies (MVBs). MVBs can be fused to lysosomes for degradation, or they can release ILVs outside the cell to form exosomes through exocytosis. Exosomes that are ultimately released between cells deliver the vesicular contents to target cells through endocytosis, direct membrane fusion, and receptor-ligand interactions.

MVBs can be fused with autophagosomes, and eventually their contents can be degraded in lysosomes, MVBs can also be directly fused with lysosomes for degradation, and the degradation products can be recycled by cells (Kalluri and LeBleu, 2020). In addition, MVBs can also be transported to the plasma membrane through the cytoskeleton and microtubule network and fused to the plasma membrane with the help of MVBs docking proteins, and through cytosolisation, ILVs in MVBs can be secreted outside the cell and become exocytosed (Colombo et al., 2014). MVBs can be classified into two categories based on their different orientations; MVBs that are degraded by fusion with lysosomes are referred to as degradative MVBs (dMVBs), and those that are fused to the plasma membrane are referred to as secretory MVBs (secretory MVBs) (Han et al., 2022). During the fusion of MVBs with the plasma membrane, the RAB family of small GTPase proteins controls the transport of intracellular vesicles, such as vesicle outgrowth, movement of vesicles through cytoskeletal roles, and the docking of vesicles to target membranes, which leads to plasma membrane fusion. Some researchers extracted different RABs involved in docking endosomes at different stages of maturation along the endosomal pathway and found that RAB11 and RAB35 can be associated with recirculation and early sorting of endosomes, whereas RAB27A and RAB27B are associated with late sorting of endosomes and secretory compartments (Stenmark, 2009; Kowal et al., 2014). In addition to the RAB family of proteins, soluble nsf attachment protein receptor (SNARE) complexes contribute to the fusion of lipid bilayers (Zylbersztejn and Galli, 2011). Specific pairing of vesicular SNAREs (v-SNAREs) with homologous target membrane SNAREs (tSNAREs) forms the SNARE complex, which drives the fusion of two opposing membranes in a zipper-like manner (Yue et al., 2020). These RAB proteins, as well as SNAREs, are thought to be required for the final fusion of MVBs with the cytoplasmic membrane (PM) (Kowal et al., 2014). Eventually MVBs fuse with the plasma membrane and release exosomes into the extracellular space (Yue et al., 2020). Exosomes can be transferred to receptor cells after release by at least three mechanisms: endocytosis, direct membrane fusion, and receptor-ligand interactions (Figure 2) (Abels and Breakefield, 2016). Ligand-receptor interactions may explain exosome-targeted biological effects, including those caused by exosome-borne growth factors, and extracellular matrix (ECM) proteins (Maas et al., 2017). However, an increasing number of studies have shown that endocytosis is the main pathway for the uptake of exosomes, which can be internalised by endocytosis mediated by lattice proteins, vesicle proteins and lipid rafts (Jadli et al., 2020). The mode of exosome uptake may also be related to the cell type and its physiological state, as well as whether or not the ligand on the exosome surface recognises the receptor on the cell surface. Ultimately, exosomes internalise the above pathways to deliver their contents to the receptor cell, thus enabling exosome messaging between cells (Abels and Breakefield, 2016; Mathieu et al., 2019).

## 4 Adipose-tissue derived exosome for wound healing

**FIGURE 3:**
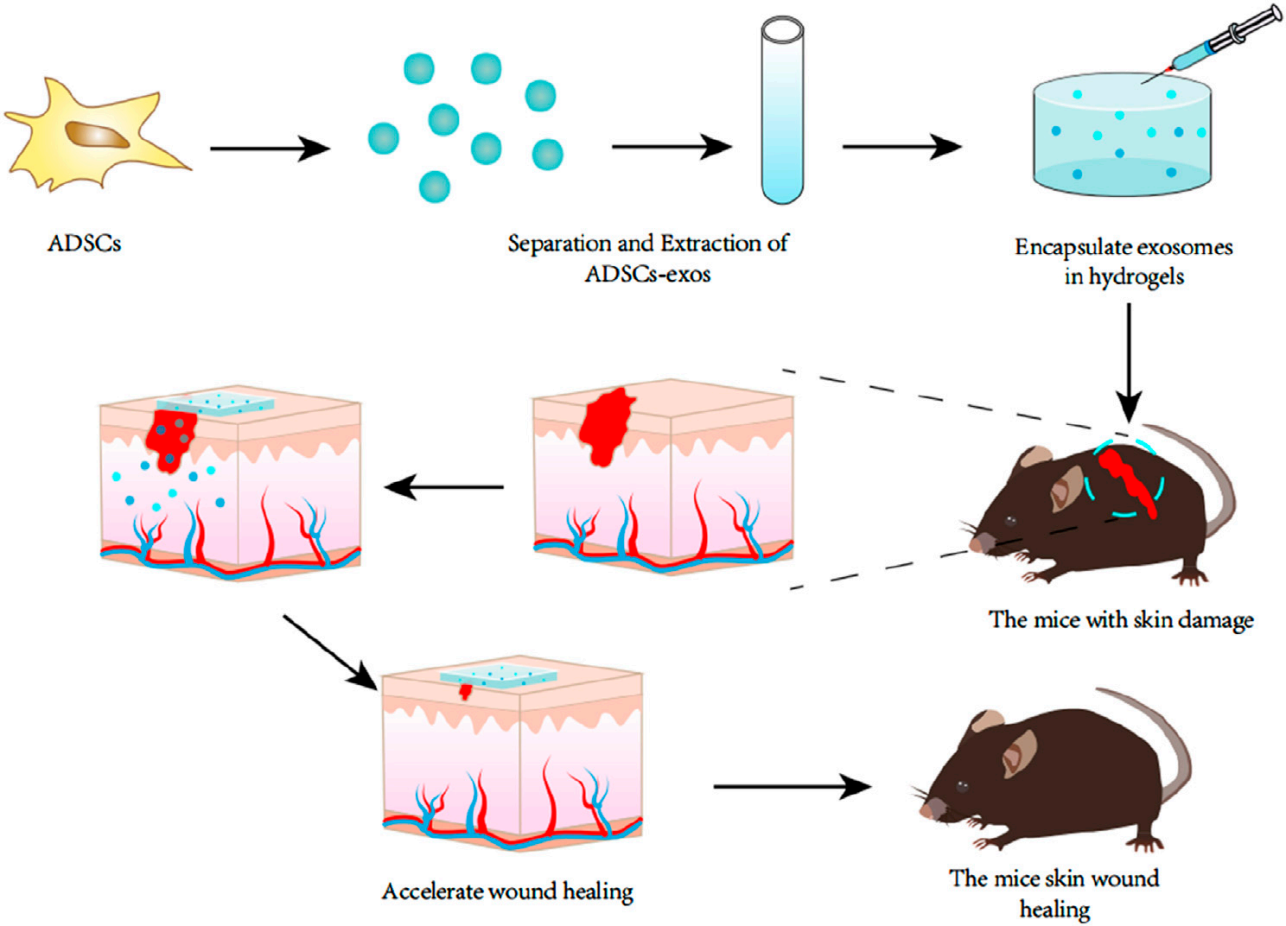
Exosomes were extracted from adipose tissue stem cells and encapsulated in a specific hydrogel, which was then locally applied to a mouse model of refractory skin injury. It was found that this hydrogel encapsulated with exosomes could promote skin wound healing better than the application of exosomes or hydrogel alone.

ADSCs-exos, as a new cell-free therapeutic strategy, has a wide range of applications in the field of regenerative medicine. A large number of studies have shown that ADSCs-exos can promote cell proliferation and tissue regeneration by activating signalling pathways or regulating gene expression to enhance or inhibit the activity of specific proteins, which provides a new therapeutic idea to promote wound healing in the clinic. Li et al. found that ADSCs-exos can promote the protein SRY-related (SRY), high-mobility-group box 9 (SOX9), Collagen I, and Collagen II (Collagen II), and the protein SRY-related (SRY), by inhibiting the expression of miR-19b. High-mobility-group box 9 (SOX9), Collagen I and Collagen III expression by inhibiting miR-19b expression, thereby promoting skin fibroblast (HSF) cell proliferation (Qian et al., 2021). The Wnt/βcatenin pathway may promote stem cell differentiation to regulate biological activity (Liu et al., 2019). Ma et al. used Western blot to detect the expression levels of Wnt and β-catenin in HaCaT cells, and β-catenin expression was elevated in the exosomes group as compared to the control group (Ma et al., 2019). In addition, SOX9 upregulated by ADSCs-exos can activate the Wnt/β-catenin pathway and promote HSF cell proliferation, migration, and invasion (Qian et al., 2021). MALAT1 serves as a transcriptional regulator of a variety of genes involved in tumour metastasis and cell migration and is involved in cell cycle regulation. He et al. found that MALAT1-containing ADSCs-exos targeting miR-124 via the Wnt/β-catenin pathway could promote cell proliferation and skin healing (He et al., 2020). Zhang et al. found that ADSCs-exos promoted elevated levels of phosphorylated Akt, Col 1, and Col 3 proteins, and that treatment of fibroblasts with Ly294002 (phosphatidylinositol kinase inhibitor) treatment of fibroblasts, Ly294002 was able to significantly inhibit exosome-induced protein level elevation (Zhang W. et al., 2018). Yang et al. found that miR-21, which enhances matrix metalloproteinase 9 (MMP-9) expression by the PI3K/AKT pathway, promotes the migration and proliferation of HaCaT cells and thus promotes wound healing (Yang et al., 2020). Ren et al. found that ADSCs-exos stimulated the AKT and ERK signalling pathways in fibroblasts resulting in significant upregulation of gene expression of proliferation markers (cyclin D1, cyclin D2, cyclin A1, cyclin A2) and growth factors (VEGFA, PDGFA, EGF, FGF2) (Ren S. et al., 2019). Furthermore, miR-19b carried by ADSCs-exos exerts its therapeutic effects on skin wounds by regulating the CCL1/TGF-β signalling axis targeting the chemokine C-C motif ligand 1 (CCL1) (L et al., 2019), and by releasing Neat1 to inhibit the expression of miR-17-5p thereby activating Ulk1-regulated autophagy (An et al., 2023). These studies suggest that ADSCs-exos can activate multiple signalling pathways to promote cell proliferation and wound healing. Zhou et al. encapsulated ADSCs-exos in Pluronic F-127 hydrogel and applied the complex topically to whole skin wounds in mice (Figure 3), and found that increasing the expression of Ki67, α-SMA, and CD31 proteins in the epithelial cells, and up-regulating the expression of the skin barrier proteins KRT1 and AQP3, the complex was more likely to promote the healing of skin wounds compared with either ADSCs-exos or PF-127 hydrogel alone. 127 hydrogel, the PF-127/ADSCs-exos complex was more able to promote skin wound healing compared with ADSCs-exos or PF-127 hydrogel alone, and this study provides a new idea for the combined application of ADSCs-exos and tissue engineering materials (Zhou et al., 2022).

Skin wounds in diabetic patients tend to be more difficult to heal than in the general population and are characterised by a prolonged skin wound healing process, leading to the development of chronic ischaemic ulcers. In contrast, ADSCs-exos are able to induce endothelial cell migration and angiogenesis by regulating several mirna, including miR-31-5p, miR-125a-5p, miR-126-3p miR-221-3p, miR-130a and miR-132 (Pomatto et al., 2021). Hypoxia, infection, reduced vascularisation and elevated oxidative stress may be key factors contributing to non-healing of chronic diabetic wounds. Wang et al. found that under hypoxic conditions, ADSCs-exos could upregulate miR-21-3p, miR-126-5p, miR-31-5p, and downregulate miR-99b and miR-146-a, genes related to wound healing. Genes, as well as activating the PI3K/Akt signalling pathway to inhibit inflammation to promote diabetic wound healing (Wang et al., 2021). Li et al. found that the transcription factor Nrf2 has an anti-oxidative stress protective effect, and the researchers applied ADSCs-exos to a diabetic rat foot ulcer model and found that ADSCs-exos could overexpress Nrf2 in a high-glucose environment thereby promoting the value-added of endothelial progenitor cells as well as angiogenesis (Li X. et al., 2018). Shiekh et al. supplemented a loaded oxygen-releasing antioxidant wound dressing OxOBand with ADSCs-exos for the treatment of diabetic wounds and found that this ADSCs-exos-added adjuvant was able to enhance collagen deposition to accelerate re-epithelialisation, as well as increase neovessel formation and decrease oxidative stress, thereby accelerating wound healing (Shiekh et al., 2020). Despite the unfavourable factors such as high glucose, high oxidative stress, and hypoxia in wounds of diabetic patients, ADSCs-exos are able to promote wound healing in this environment by modulating a variety of small molecule nucleic acids, and this study provides a new therapeutic idea for treating diabetic patients’ wound healing in the clinic. The current study only focuses on skin wounds caused by simple diabetes. Future studies should also consider the effect of diabetes type (type 1 and type 2), chronic wound and patient variability on the treatment of ADSCs-derived exosomes, which will be critical for clinical translation.

ADSCs-exos also has a wide range of applications in the field of aesthetic medicine. Kwon et al. treated acne scars with a combination of fractional carbon dioxide laser (FCL) and ADSCs-exos, and found that ADSCs-exos could promote skin recovery and reduce the appearance of post-procedural erythema after ablation of FCL by increasing a variety of anti-inflammatory factors and regenerative growth factors. Skin recovery and reduced the appearance of post-procedural erythema by increasing multiple anti-inflammatory factors and regenerative growth factors (Kwon et al., 2020). Trzyna et al. found that ADSCs-exos not only reduced inflammation and apoptosis to repair flaps, but also prolonged the survival of vascularised composite allografts after transplantation by down-regulating CD4^+^ T and Th1 cells, and up-regulating Tr1 and Treg cells, by which the success rate of allograft implantation could be further improved (Trzyna and Banaś-Ząbczyk, 2021). Reducing healing time and scar formation in soft tissue trauma recovery is a pressing clinical need today. Current conventional methods to accelerate healing and reduce scar formation include skin grafting, laser therapy and topical application of some growth factors or gene therapy. However, these methods may lead to adverse consequences such as atrophic scars, pigmentation abnormalities, and skin necrosis. In the early stage of wound healing, ADSCs-exos increased the production of collagen types I and III to promote wound healing, whereas in the later stage, ADSCs-exos inhibited collagen expression and reduced scar formation (Hu et al., 2016). This conclusion is also supported by a study that ADSCs-exos play different roles at different stages of wound healing and scarring phase. Li et al. found that ADSCs-exos could regulate the ratio of fibroblast Col-III to Col-I, TGF-3 to TGF-1, and MMP3 to TIMP-1 during the late healing phase, shifting fibroblasts to an endogenous state and inhibiting fibroblast differentiation thereby reducing scarring (Li C. et al., 2022). This mechanism may be that ADSCs-exos inhibit the expression of Smad3 and Notch-1 proteins as well as transforming growth factor β2 (TGF-β2) in KFs, promote the expression of TGF-β3 as well as inhibit the expression of collagen type I (COL-1), collagen type III (COL-3), fibronectin (FN) and α-smooth muscle actin (α-SMA) gene and protein expression (Li J. et al., 2022). In addition, miR-192-5p is also a regulatory mode of ADSCs-exos to reduce the level of proliferative scar fibrosis (Li Y. et al., 2021). These studies demonstrated that ADSCs-exos also plays a great potential in inhibiting scar formation in the medical aesthetic field, whether it is skin reimplantation, allograft or scar repair, ADCSs-exos can promote the repair of damaged skin by regulating or activating specific signalling pathways.

## 5 Musculoskeletal regeneration

There is still a high clinical demand for effective bone regeneration therapy. Autologous and allogeneic bone grafts may lead to various complications, and the safety of cellular therapy needs to be further demonstrated. Exosomes have been found to have a possible role in the regeneration of the musculoskeletal system (Qin et al., 2016). ADSCs-exos can be used as a biomimetic tool to regulate and induce the differentiation of stem cells to osteoblasts. The osteogenesis-promoting effects of ADSCs-exos have been attributed to four main mechanisms; reduction of apoptosis in ischemic and necrotic microenvironments, recruitment of MSCs and promotion of their proliferation, promotion of angiogenesis, and direct promotion of osteogenic differentiation of MSCs (Liu et al., 2017; Zhang et al., 2016; Qi et al., 2016). Chen et al. found that miR-375 could be overexpressed in adipose stem cells and enriched in exosomes, and these exosomes carrying miR-375 stimulated mRNA expression of relevant osteogenic genes (Chen S. et al., 2019). Insulin-like growth factor binding protein 3 (IGFBP3), an inhibitor of osteoblast differentiation, inhibits bone morphogenetic protein 2 (BMP - 2) signalling, and miR-375 can downregulate IGFBP3 expression (Chen S. et al., 2019; Eguchi et al., 2018). Lu et al. treated ADSCs-exos with tumour necrosis factor-α (TNF-α) and found that ADSCs-exos were able to promote the proliferation and osteogenic differentiation of primary osteoblasts (HOBs), and this osteogenic capacity was further enhanced after tumour necrosis factor-α (TNF-α) treatment. Pretreated exosomes showed elevated Wnt-3a content, and it was found that inhibition of the Wnt signalling pathway led to a significant reduction in the level of osteogenic gene expression, so this mechanism may provide a mechanism for the enhancement of primary progenitor generation by TNF-α pretreatment through increasing Wnt-3a content in exosomes proliferation of osteoblasts (Lu et al., 2017). An experiment comparing the effects of exosomes from different tissue stem cell sources on cartilage and bone regeneration analysed the role of ADSCs-exos, BMSCs- exos and SMSCs - exos in promoting cartilage differentiation *in vitro*, and found that the gene for the hypertrophy-associated marker collagen type X (Col X) was markedly downregulated in all three types of exos-treated BMSCs, but was most markedly downregulated in the ADSCs-exos group. In turn, hypertrophy adversely affects the chondrocyte differentiation process, suggesting that ADSCs-exos is more advantageous for chondrogenic differentiation in obese patients. This study also demonstrated that ADSCs-exos induced BMSCs into chondrogenesis more significantly. BMSCs treated with ADSCs-exos could more significantly express proteins COL I, COL II, and SOX9, which can promote cartilage matrix synthesis, and ADSCs-exos showed the best bone-related matrix production performance among all groups, demonstrating better bone regeneration ability of ADSCs-exos (Li Q. et al., 2021).

Parental cell pretreatment of exosomes can enhance the regenerative potential of exosomes for bone defects. With the development of various biomaterials, biomaterial-assisted exosomes have become a promising strategy for bone regeneration (Lu et al., 2023). ADSCs-exos treated with osteogenesis-directed culture solution could upregulate the mRNA expression of relevant osteogenic genes, such as RUNX2, ALP, COL1A1, in addition to significantly promoting the osteogenic differentiation of BMSCs by enhancing ALP activity, and extracellular mineralisation nodules. Under certain conditions, BMSCs were immobilised on polydopamine-coated PLGA/pDA scaffolds, and this composite exosome PLGA/pDA scaffolds could consistently and effectively promote bone regeneration (Li W. et al., 2018). Chen et al. integrated miR-375-ADSCs-exos into a hydrogel and applied it to a rat model, and found that bone regeneration was enhanced in rats with cranial bone defects (Chen S. et al., 2019). Li et al. used tissue-specific decellularised extracellular matrix (dECM) and ADSCs-exos as materials for a novel biomimetic dual-network hydrogel scaffold prepared by 3D printing, and by monitoring cartilage-specific genes, it was found that the expression levels of genes including aggregated proteoglycan (ACAN), collagen type II (COL II) and sy -box transcription factor 9 (SOX9) were significantly upregulated in both the Hydrogel-DCM and Hydrog-cultured rBMSCs were significantly upregulated. It was also found that 3d-printed microenvironment-specific heterogeneous bilayer scaffolds effectively accelerated the simultaneous regeneration of cartilage and subchondral bone tissue in a rat OC defect model (Li Q. et al., 2023).

ADSCs-exos not only have pro-regenerative, anti-inflammatory and anti-apoptotic properties in orthopaedic diseases, but have also been found to play a key therapeutic role in muscle tissue regeneration. Figliolini et al. found that ADSCs-exos had a role in driving myocyte proliferation and differentiation in an *in vitro* ischaemia model. In addition researchers found that ADSCs-exos showed anti-apoptotic effects both *in vivo* and *ex vivo*, and that ADSCs-exos carried a variety of pro-angiogenic mrna and NRG1. In contrast, NRG1 is associated with muscle protection, vascular growth and inflammatory cell recruitment. The involvement of ADSCs-exos in vascular growth and muscle regeneration was confirmed by bioinformatics analysis of 18 molecules detected in ADSCs-exos (Figliolini et al., 2020). In a rat model of severe rotator cuff tear (MRCT), ADSCs-exos were injected into the lesion, and when compared with the control group group, it was found that not only muscle atrophy, fatty infiltration, inflammation, and vascularisation could be prevented in the ADSCs-exos group, but also muscle fibre regeneration and biomechanical properties of tendon sheaths of the rats treated with ADSCs-exos were significantly improved (Wang et al., 2019). Fu et al. found that ADSCs-exos could promote the proliferation and differentiation of tendon-derived stem cells (tdsc) through the paracrine pathway (Fu et al., 2021). In a controlled trial, Doan et al. found that the expression of genes Flk1, Vwf, Ang1, Tgfb1, Myod, and Myf5, which promote angiogenesis and muscle remodelling, was increased 4-8-fold in the ADSCs-exos group compared to the control group, by intravenously injecting ADSCs-exos into a mouse model of hindlimb ischemia compared to the placebo group (Doan et al., 2023). Mitchell et al. tested the regenerative effects of extracellular vesicles using a model of skeletal muscle injury and found that soluble factors secreted by ADSCs-exos promoted muscle regeneration *in vitro* and *in vivo* in a highly synergistic manner (Mitchell et al., 2019). Zhu et al. summarised the possible mechanisms of ADSCs-exos in muscle regeneration as follows: ADSCs-exos promotes the classical Wnt pathway to dominate the myogenic function of satellite cells, activates the ERK1/2 and JNK MAPK pathways to stimulate myoblast proliferation at an early stage, assists the PI3K/Akt pathway to promote protein synthesis and myofibre hypertrophy, and promotes the JAK2/STAT2/STAT3 pathway to advance the myoblast differentiation process (Zhu et al., 2022). Although there are fewer studies on the regeneration of the musculoskeletal system by ADSCs-exos, it can be shown from the above studies that ADSCs-exos play an important role in bone and muscle regeneration, and that these exosomes can stimulate the expression of relevant genes or activate specific signalling pathways to promote bone and muscle regeneration. In addition, these effects and advantages of exosomes can also be used to combine with biological tissue engineering to continue to play its role. These studies provide a strong theoretical basis and good innovative ideas for the subsequent application of exosomes in clinical treatment.

## 6 Cardiovascular regeneration

Existing studies suggest that ADSCs-exos may have a role in promoting cardiovascular regeneration, improving cardiovascular fibrosis, and preventing and reducing the extent of atherosclerosis. The mechanism of action may be through the induction of signalling pathways by mirna or small molecule proteins carried by ADSCs-exos. Insulin resistance is a known risk factor for cardiovascular disease, and it has been found that ADSCs-exos can reduce systemic insulin resistance. Injection of ADSCs-exos into the peritoneal cavity of a high-fat mouse model revealed that these mice showed improved glucose tolerance and insulin sensitivity, as well as reduced levels of serum triglycerides and total cholesterol *in vivo*, which reveals a possible role of ADSCs-exos in the prevention and treatment of cardiovascular-related metabolic diseases (Constantin et al., 2020; Ormazabal et al., 2018). Fan et al. measured increased markers of vascular endothelial cell activation VEGF, ET-1, VCAM-1, and ICAM-1 as well as decreased markers of inflammation CRP, IL-6, and TNFα, and also found reductions of atherosclerotic plaques in mice after an intravenous infusion of ADSCs-exos in atherosclerotic rats (Fan et al., 2019). Deng et al. found that ADSCs-exos were able to prevent oxidative stress-induced apoptosis in cardiomyocytes in in vitro experiments. In addition researchers injected ADSCs-exos intravenously into an animal model of myocardial infarction and found that ADSCs-exos were able to induce M2-type macrophage polarisation and immune cell transport, culminating in improved haemodynamic outcomes observed with echocardiography in these animals (Deng et al., 2019). Xing et al. investigated the effect of ADSCs-exos on the differentiation of ADSCs to endothelial cells and found that ADSCs-exos may reduce endothelial cell apoptosis by decreasing the expression of miR-324-5p, a potential marker and effector of atherosclerosis (Xing et al., 2020b). Cui et al. found that ADSCs-exos could protect cardiomyocytes from ischaemia-reperfusion injury by activating the Wnt/β-catenin signalling pathway (Cui et al., 2017). Lou et al. found that ADSCs-exos overexpressing miR −126 reduced cardiac fibrosis and pro-inflammatory cytokine levels while promoting angiogenesis in a rat model of myocardial infarction and a model of hypoxia-induced H9c2 cardiomyocyte injury (Luo et al., 2017). In addition, Liu et al. found that mir -93-5p-modified ADSCs-exos exhibited a stronger protective effect against acute myocardial infarction-induced myocardial injury compared to unmodified ADSCs-exos, which was mediated by the reduction of atg7-mediated autophagy and inhibition of TLR4 (Liu et al., 2018). These studies can demonstrate that ADSCs-exos can regulate lipid levels *in vivo* and improve atherosclerosis as well as protect the myocardium to reduce myocardial fibrosis.

The very first studies found that ADSCs have the ability to differentiate towards endothelial and smooth muscle cells, which can be used in the preparation of artificial blood vessels. The use of uniaxial mechanical strain at a frequency of 1 Hz and TGF-β1 stimulation allowed the differentiation of ADSCs towards smooth muscle cells (Lee et al., 2007). Vascular endothelial growth factor (VEGF) has the ability to induce the differentiation of stem cells in the direction of vascular cells, and Rehman et al. showed that ADSCs cultured under hypoxic conditions were found to secrete a wide range of biologically active angiogenic and anti-apoptotic growth factors, and the proliferation of endothelial cells and the inhibition of their apoptosis are essential for the growth of new blood vessels, so that the secretion of these growth factors by ADSCs may promote angiogenesis (Kranz et al., 2000; Rehman et al., 2004). Yu et al. found that mirna-engineered modified ADSCs were able to promote cell proliferation and vascular regeneration (Yu et al., 2020). These studies imply that ADSCs play a role in cardiovascular regeneration. Zhu et al. demonstrated *in vitro* and *in vivo* that ADSCs-exos promoted angiogenesis by promoting VEGF secretion from vascular endothelial cells through the let-7/argonaute 1 (AGO1)/VEGF signalling pathway (Zhu et al., 2020). Xu et al. observed that some pro-angiogenic mirna was enriched in ADSCs-exos, which could be exocytosed and internalised into human umbilical vein vascular endothelial cells and could act as paracrine signals to promote angiogenesis (Xu et al., 2019). Lou et al. found that the area of myocardial infarction and fibrosis after acute infarction was reduced with miR-126-enriched ADSCs-exos. In an *in vivo* animal model, recovery of perfusion in ischaemic muscle was significantly increased by intramuscular injection of miR-126-enriched ADSCs-exos, suggesting that ADSCs-exos may reduce the area of myocardial infarction and fibrosis after acute infarction by restoring vascular perfusion (Luo et al., 2017). ADSCs-exos from a porcine model of hyperlipidaemia was found to alter gene expression in cardiac fibroblasts (CF) in a simulated ischaemic environment, resulting in the upregulation of cardiomyocyte-specific transcription factors and healing markers α- sma, such as GATA4, Nkx2.5, IRX4, and TBX5, in CF, and the finding that the biomarkers in fibroblasts vimentin, FSP1, podoplanin and cardiac biomarkers troponin- i and connexin-43 were downregulated. With these markers we can learn that ADSCs-exos can modulate specific genes to improve healing after myocardial ischaemic injury (Thankam et al., 2023). Song et al. found that ADSCs-exos transfected with miR-210 regulated protein tyrosine phosphatase 1B (PTP1B) and death-associated protein kinase 1 (DAPK1) to inhibit apoptosis and promote cardiovascular regeneration through the inhibition of Ephrin-A3 (EFNA3) under hypoxic conditions (Song et al., 2021). Lelek et al. found that ADSCs-exos had anti-inflammatory, anti-apoptotic reduction and pro-angiogenic effects by injecting them into ischaemic tissues (Lelek and Zuba-Surma, 2020). The above studies provide a rationale for the role of ADSCs-exos in promoting cardiovascular regeneration, preclinical evidence suggests that the bioactive factors carried by exosomes can promote the proliferation of vascular endothelial cells and neovascularisation. The application of ADSCs-exos in cardiovascular disease is promising, and future studies need to further explore the mechanisms of these effects and how their therapeutic efficacy can be applied in the clinic, but a number of challenges such as safety and efficacy will need to be addressed before they can be applied in the clinic.

## 7 Nervous system regeneration

Neurological injury caused by trauma or disease has always been a clinical challenge, and whether neuronal cells can be regenerated after injury is currently a focus of academic debate. Peripheral nerve injury (PNIs) is a common neurological disease, and it has been shown that ADSCs can play a regenerative therapeutic role in PNIs by secreting growth factors or promoting myelination through differentiated Schwann cells (SCs). ADSCs-exos may be the key to treating PNIs, and cell-free therapeutic studies of ADSCs-exos have provided new breakthroughs in neuronal cell regeneration after neurological injury (Ju et al., 2023; Jiang et al., 2022). Bucan et al. found that ADSCs-exos promoted Schwann cell proliferation, increased the expression of the cell cycle protein Ki67, and enhanced the axon length of dorsal root ganglion (DRG) neurons. In addition, researchers have confirmed the presence of nerve growth factors such as brain-derived neurotrophic factor (BDNF), insulin-like growth factor-1 (IGF-1), nerve growth factor (NGF), fibroblast growth factor-1 (FGF-1), and glial cell-derived neurotrophic factor (GDNF) in ADSCs-exos (Bucan et al., 2019). Ching et al. found that ADSCs-exos transfer the same RNAs as Schwann cell-derived exosomes, and that these RNAs play an important role in promoting neurite growth (Ching et al., 2018). In PNIs model rats, ADSCs-exos reduced apoptosis in SCs after PNIs by up-regulating the expression of anti-apoptotic Bcl-2 mRNA and down-regulating the expression of pro-apoptotic Bax mRNA (Liu et al., 2020). Yang et al. found that ADSCs-exos may also inhibit the expression of chromosome 10 deletion phosphatase and tensin homologue (PTEN) through miRNA-22-3p, activating the phosphorylation of the AKT/mTOR axis to promote the proliferation and migration of SCs cells, and additionally it was found that ADSCs-exos could promote the growth of dorsal root ganglion (DRG) axons (Yang et al., 2022). Ren et al. found that ADSCs-exos modified with mir −133b significantly promoted neurological recovery in spinal cord injured animals by affecting the axonal regeneration-related signalling pathway and the expression of related proteins NF, GAP-43, GFAP and MBP (Ren Z. W. et al., 2019). ADSCs can differentiate towards the Schwann cell phenotype and exosomes derived from such differentiated cells have been found to secrete more neurotrophic and other growth factors (Liu et al., 2022). In a rat sciatic nerve severance model, ADSCs-exos internalised by Schwann cells significantly promoted SCs proliferation, migration, myelination and secretion of neurotrophic factors by up-regulating the corresponding genes, thereby promoting peripheral nerve regeneration (Chen J. et al., 2019). These findings suggest that exosomes promote Schwann cell proliferation for neuronal regeneration through the regulation of growth factors and miRNAs. Pigment epithelium-derived factor (PEDF), a 50-kDa secreted glycoprotein, has been shown to have protective effects on cultured cortical neurons by inhibiting oxidative stress and apoptosis. ADSCs-exos modified by PEDF strongly inhibit neuronal apoptosis through caspase-dependent (caspase-9 and caspase-3) pathways (Huang et al., 2018; Sanchez et al., 2012). After neural injury, macrophage-induced autophagy occurs in the peripheral environment of innervated neurons, affecting the regulation of inflammatory responses. Whereas the immunosuppressant FK506 promotes nerve regeneration after nerve crush injury and allogeneic nerve transplantation, the combination of FK506 and ADSCs in a mouse sciatic nerve crush injury model revealed that ADSCs-FK506-exos significantly reduced autophagy in segmental macrophages after spinal cord injury (Kuo et al., 2021). However, another study in which ADSCs were pretreated with FK506 found that the exosomes secreted by both ADSCs pretreated with FK506 and untreated ADSCs did not show significant differences in their nerve regeneration-promoting effects, but histone deacetylases (HDACs), β-amyloid A4 (APP), and integrin β1 (ITGB1) detected in the exosomes of both were involved in the promotion of nerve regeneration (Rau et al., 2021). This suggests that FK506, although not able to enhance the effects of ADSCs-exos on nerve regeneration, can act synergistically with ADSCs-exos and together act as a neuroprotecting and nerve regenerating agent. Yin et al. found that the autophagy level and the expression of nuclear transporter protein subunit α2 (Kpna2) in Schwann cells were significantly increased after sciatic nerve injury through peripheral nerve injury rat model experiments. Researchers found that ADSCs-exos could inhibit SCs autophagy by down-regulating Kpna2 via miRNA-26b through *ex vivo* experiments (Yin et al., 2021). In addition, Farinazzo et al. found *in vitro* that low doses of ADSCs-exos were able to exert a protective effect on neuronal cells exposed to oxidative damage by H2O2, and that their mechanism of action may exert an anti-apoptotic effect by inhibiting the H2O2-induced apoptotic cascade response in neuronal cells and by increasing neuronal cell viability. Moreover, this study identified ADSCs-exos as a source of relevant regenerative factors that may modulate the microenvironment in neuroinflammatory and neurodegenerative diseases, thereby promoting neuronal regeneration (Farinazzo et al., 2015). These studies suggest that ADSCs-exos can promote neuronal regeneration by carrying associated factors and can also be neuroprotective by reducing phagocytosis by macrophages or inhibiting oxidative damage.

ADSCs-exos can play a role in neuroprotection and neuroregeneration in neurodegenerative diseases. Alzheimer‘s disease (AD) is a neurological disorder characterised by cognitive deficits and pathologically characterised by the accumulation of β-amyloid plaques and progressive neuronal death in the hippocampus and cerebral cortex (Molinuevo et al., 2018). Lee et al. found that ADSCs-exos reduced β-amyloid levels in AD neuronal cells as well as apoptosis in AD neuronal cells, and furthermore, the researchers found that ADSCs-exos were able to promote axonal growth in neural stem cells (Lee et al., 2018). By applying ADSCs-exos transfected with miRNA-22 to a mouse model of Alzheimer’s disease (AD), Zhai et al. found that it could inhibit cellular pyroptosis to reduce the release of inflammatory factors to play a therapeutic role in AD (Zhai et al., 2021). Neurotrophic factor-3 (NT-3) is an important neurotrophic factor in the process of peripheral nerve regeneration. ADSCs-exos were used as a carrier of NT-3 mRNA, loaded in nerve-guided catheters and bridged to a rat model of deficient sciatic nerve, which ultimately revealed that NT-3 mRNA promotes nerve regeneration mediated by ADSCs-exos (Yang et al., 2021). These studies suggest that ADSCs-exos can also be used to treat neurodegenerative diseases or as a vehicle to promote nerve regeneration and treat nerve injuries. Based on the above studies ADSCs-exos is expected to provide a new therapeutic strategy for the clinical treatment of nerve injuries and the promotion of nerve regeneration. Furthermore, when using ADSCs-exos to promote neural regeneration, the following issues should also be taken into consideration, such as penetration of the blood-brain barrier, targeted delivery to the central nervous system, dosing/bioavailability, and standardization of exosome purity. These issues will be a series of challenges that ADSCs-exos therapy for nerve injury faces in its transition to clinical application.

## 8 The clinical application of adipose-derived stem cell exosomes

The therapeutic potential of ADSCs-exos in a variety of diseases has been unearthed in recent years through the study of ADSCs-exos. ADSCs-exos carry a variety of genetic material and proteins that enable them to participate in a wide range of biological processes, including cell proliferation, migration, and apoptosis, modulation of the immune and inflammatory response, regulation of osteoblast metabolism, promotion of angiogenesis, and influence on tumour growth [(Hong et al., 2019)]. Moreover, the therapeutic effects of ADSCs-exos have been demonstrated in different disease models, such as promoting diabetic skin wound healing, reducing scars after wound repair, improving fat grafting, regeneration of bone and muscular system, treating myocardial infarction and ischemia/reperfusion injuries, and delaying neurodegeneration (Table 1). This augurs well for the possible clinical application of ADSCs-exos as a cell-free therapy.

**TABLE 1:** Adipose-derived stem cell exosomes promote the regeneration of various tissues by regulating related genes and signaling pathways.

Gene	Signal pathway	Reproduction organization
miR-19b, SOX9	Wnt, β- catenin, PI3K, AKT, ERK	epithelial tissue
miR‐375, RUNX2, ALP, COL1A1	Wnt	bone tissue
Flk1, Vwf, Ang1, Tgfb1, Myod, Myf5	Wnt, ERK1/2, JNK MAPK, PI3K/Akt, JAK2/STAT2/STAT3	muscular tissue
miR-324-5p, miR -93-5p	Wnt/β-catenin	Cardiovascular
Bcl-2 mRNA, miR-22-3p, miR −133b, miR-26b	AKT/mTOR	Nervous tissue

One of the most challenging complications of diabetes is delayed wound healing, and hypoxia, reduced vascularisation, elevated oxidative stress and infection are key factors in the non-healing of chronic diabetic wounds (Shiekh et al., 2020; Greenhalgh, 2003). Wang et al. demonstrated that ADSCs-exos could promote wound healing in diabetic mice by promoting angiogenesis, fibroblast proliferation and migration, and collagen synthesis (Wang et al., 2020). ADSCs-exos express antioxidant receptors (Nrf2) that promote angiogenesis to accelerate diabetic wound healing (Li X. et al., 2018). It has also been found that ADSCs-exos play different roles at different stages of wound healing, with ADSCs-exos regulating the ratio of fibroblast Col-III to Col-I in the late stages of healing to inhibit fibroblast differentiation as well as decreasing the level of proliferative keloid fibrosis through specific genes (Li C. et al., 2022; Li Y. et al., 2021). In terms of improving fat grafting, ADSCs-exos can increase the retention of fat graft volume by stimulating angiogenesis and modulating inflammatory responses. In addition, it was found that hypoxia-treated ADSCs-exos had a higher ability to promote angiogenesis in fat grafting. This ability to retain grafted fat volume greatly improves the success of fat grafting in medical aesthetics (Mou et al., 2019; Han et al., 2019). Based on the biocompatibility and cellular targeting of exosomes can be loaded into biological tissue materials, perpetuating the release of exosomes to enhance wound healing (An et al., 2021). Shilan et al. were able to significantly promote wound healing and local angiogenesis in an *in vivo* study using exosomes loaded in sodium alginate gel as a bioactive scaffold (Shafei et al., 2020). These studies have demonstrated the therapeutic ability of ADSCs-exos in skin wound healing.

ADSCs-exos in the regeneration of the musculoskeletal system, treatment with ADSCs-exos prevented muscle atrophy, fatty infiltration, inflammation, and vascularisation in a rat massive rotator cuff tear (MRCT) model, resulting in significant improvements in tendon sheath muscle fibre regeneration and biomechanical properties (Wang et al., 2019). Li et al. could accelerate the repair of critical skull defects in mice by combining ADSCs-exos with polylactic acid-hydroxyacetic acid copolymer (PLGA) scaffolds and showed that PLGA scaffolds combined with exosomes recruited more MSCs *in vivo* by immunofluorescence staining (Li W. et al., 2018).

Neurodegenerative diseases such as Alzheimer’s disease (AD), Huntington’s chorea (HD), and Parkinson’s disease (PD) are commonly characterised by progressive deterioration and death of nerve cells. ADSCs-exos were found to reduce β-amyloid levels in AD neuronal cells as well as reduce apoptosis in AD neuronal cells (Lee et al., 2018). Enkephalinase (NEP) is a therapeutic target for AD, and Katsuda et al. demonstrated that ADSCs-exos were able to secrete enzymatically active NEP, co-cultured ADSCs with neuroblastoma N2a cells, and ultimately found that ADSCs-exos led to a reduction in intracellular and extracellular Aβ levels (Katsuda et al., 2013). In a mouse model of amyotrophic lateral sclerosis (ALS) with increased superoxide dismutase (SOD-1) aggregation, ADSCs-exos were able to reduce SOD-1 aggregation and normalise aberrant mitochondrial protein levels as well as normalise PGC-1α and p-CREB/CREB ratios in ALS mutants (Lee et al., 2016). These studies were able to demonstrate the therapeutic potential of ADSCs-exos in neurodegenerative diseases.

The occurrence of ischaemia-reperfusion in any tissue or organ may lead to the generation of reactive oxidative stress and even lead to organ failure causing severe irreversible damage to the body. Some studies have shown that ADSCs-exos could be a new tool as a novel cell-free therapy to treat ischaemia-reperfusion injury (Yapca et al., 2013). Cui et al. found that ADSCs-exos could exert anti-apoptotic and pro-survival effects on cardiomyocytes by activating the Wnt/β-catenin signalling pathway, thereby protecting cardiomyocytes from ischemia-reperfusion injury (Cui et al., 2017). Lin et al. found that ADSCs-exos protected the kidneys from acute ischaemia-reperfusion injury and attenuated inflammatory response, oxidative stress and deterioration of renal function (Lin et al., 2016). Huang et al. found that ADSCs-exos modified with pedf were able to activate autophagy and inhibit neuronal apoptosis to attenuate cerebral ischaemia or reperfusion injury (Huang et al., 2018). In flaps after ischaemia-reperfusion injury, ADSCs-exos promote neovascularisation and attenuate inflammatory response and apoptosis (Bai et al., 2018). These studies demonstrated the therapeutic effects of ADSCs-exos on ischaemia-reperfusion injury in different organs, and ADSCs-exos cell-free therapy may become a valuable tool for the clinical treatment of ischaemia-reperfusion injury.

## 9 Conclusion

ADSCs-exos has an extremely outstanding performance in various tissue regeneration fields. It can inherit abundant bioactive molecules, such as proteins, nucleic acids and small molecule signalling molecules, from parent cell ADSCs, and carry these molecules to participate in cell-to-cell communication and a variety of biological processes in order to promote cell proliferation and tissue regeneration. ADSCs-exos, as a cell-free treatment, is also able to overcome the drawbacks of cellular therapies such as cellular immune rejection, which opens up the possibility of alternative cellular therapies. In addition, ADSCs-exos are also capable of synergising therapeutic effects by pre-treating the culture so that it carries specific small molecule nucleic acids, or acting as drug carriers for targeted drug transport to target tissues. However, there are still many difficulties in applying ADSCs-exos in the clinic. Although adipose tissue is widely available and easy to obtain, there are still technical bottlenecks in isolating, extracting and purifying exosomes and producing them on a large scale, and the current production methods are complicated, which still can’t realise large-scale production. Moreover, the heterogeneity of the donors, variations in culture conditions (such as differences in serum batches), and the lack of standardization in the induction protocols all contribute to fluctuations in the production and function of exosomes across different batches. ADSCs-exos are also relatively unstable and prone to lose biological activity during storage and transport, and further research and development of effective preservation and transport methods are needed. It has been shown that artificial micro- and nano vesicles are able to possess a higher proliferation capacity than natural ADSCs-exos (Kim et al., 2017), which provides a good idea for the preparation of exosomes, although a large number of studies are needed to prove whether artificial nano vesicles are superior to natural exosomes in all aspects or in the treatment of a variety of diseases. An additional challenge that prevents ADSCs-exos from being used in the clinic is that it is still not possible to determine a specific safe dose for use in humans, and further research is needed to determine the safe range of therapeutic measures. We also need to expand our knowledge of ADSCs-exos over a long period of time, with large samples, and gradually moving from animal to clinical trials. At the regulatory and production levels, the ambiguity of exosome classification, the absence of regulatory frameworks, and the challenges in selecting clinical indications (such as the design of endpoint indicators) collectively constitute barriers to industrialization. To break through these bottlenecks, interdisciplinary collaboration is required, encompassing the elucidation of basic mechanisms, standardization of processes, the development of innovative analytical methods, and the establishment of regulatory science cooperation, in order to facilitate a substantive leap from laboratory to clinical application of ADSC-Exos. Although there are some challenges in the application of ADSCs-exos to the clinic, ADSCs-exos has a broad prospect as a cell-free therapy in clinical application. With the advancement of technology, the preparation method and scale-up production technology of exosome will be gradually improved to make it more suitable for clinical application. Meanwhile, the biological activity and stability of exosomes will be further improved to enhance their effectiveness and safety in therapy. In the future, ADSCs-exos are expected to be applied to disease regeneration therapy, opening up new avenues for cell-free treatment.
